# Identifying key risk factors for thrombocytopenia in patients undergoing radical chemoradiotherapy for nasopharyngeal cancer: a retrospective study

**DOI:** 10.3389/fonc.2026.1752387

**Published:** 2026-03-02

**Authors:** Qiongling Huang, Xinyuan Luo, Baoling Li, Ming Lu, Xiaofang Chen, Enhui Qiu

**Affiliations:** Department of Otolaryngology, The Second Affiliated Hospital of Fujian Medical University, Quanzhou, Fujian, China

**Keywords:** chemoradiotherapy, diabetes mellitus, hypertension, nasopharyngeal carcinoma, risk factors, thrombocytopenia

## Abstract

**Introduction:**

In this study, we investigated the risk factors for thrombocytopenia in patients undergoing radical chemoradiotherapy for nasopharyngeal carcinoma (NPC).

**Methods:**

We retrospectively analyzed clinical data and laboratory indicators of 318 patients initially diagnosed with NPC who received radical chemoradiotherapy at our institution between January 2013 and May 2024. The patients were stratified into three groups based on the severity of post-chemoradiotherapy thrombocytopenia. Clinical parameters and laboratory indices were then systematically compared among these groups.

**Results:**

Platelet counts before and after chemoradiotherapy differed significantly (Z = −15.421; *p* < 0.001). No significant differences were observed among the groups in age, sex, hypertension, diabetes, alcohol consumption, or T, N, and M stages (all, *p*> 0.05). However, smoking status differed significantly (*p* < 0.001). Logistic regression identified smoking (odds ration [OR] = 3.393; 95% confidence interval [CI], 1.88–6.123; *p* < 0.001) and preoperative platelet count (OR = 0.993; 95% CI, 0.989–0.997; *p* < 0.001) as independent risk factors for post-chemoradiotherapy thrombocytopenia.

**Conclusion:**

Smoking and low baseline platelet counts were independent risk factors for thrombocytopenia following radical chemoradiotherapy in patients with NPC. Therefore, dynamic monitoring of platelet levels in smokers and in those with low preoperative counts is crucial throughout treatment.

## 1 Introduction

Nasopharyngeal cancer (NPC) originates from the nasopharyngeal epithelium and is a prevalent malignant tumor in the head and neck region, particularly in Southeast Asia and southern China (1). Conversely, NPC is rare in Western countries. The etiology of NPC primarily involves Epstein–Barr virus infection, along with genetic and environmental factors (2). Due to its deep anatomical location and the subtleness of early symptoms, 80–90% of NPC cases are diagnosed at intermediate or advanced stages (3, 4). Additionally, the risk of metastasis is significantly elevated in these stages compared with other head and neck squamous cell carcinomas (5).

Approximately 60% of patients with NPC are diagnosed due to a neck mass, which typically indicates lymph node metastasis. According to the National Comprehensive Cancer Network guidelines, treatment of locoregionally advanced NPC (stages T1 with N1–N3 and T2–T4 with any N) involves concurrent chemoradiotherapy with either adjuvant chemotherapy or induction chemotherapy followed by radiotherapy or chemoradiotherapy (2). Chemoradiotherapy is effective in treating early-stage and non-metastatic NPC (6). However, approximately 20–30% of patients with advanced NPC experience treatment failure, mainly due to recurrence and metastasis (7, 8). Only 65% of these patients achieve a five-year survival rate (9). Locally advanced NPC is typically managed with concurrent chemoradiotherapy, marking a significant therapeutic advancement (10). Nonetheless, patients with malignant tumors often experience varying degrees of thrombocytopenia after undergoing chemotherapy or chemoradiotherapy. While concurrent chemoradiotherapy is the standard treatment for locoregionally advanced nasopharyngeal carcinoma, the hematologic toxicity it induces, particularly thrombocytopenia, remains a significant limiting factor impacting the successful implementation of therapy and patient prognosis. Although concurrent chemoradiotherapy is the standard treatment for locoregionally advanced nasopharyngeal carcinoma, the hematologic toxicity it induces, particularly thrombocytopenia, remains a key limiting factor affecting the successful implementation of therapy and patient prognosis.

Meanwhile, existing studies have demonstrated that the incidence of thrombocytopenia is substantial among NPC patients undergoing CCRT or induction chemotherapy. For example, researchers such as Lee have reported, and previous clinical trials have indicated, that the incidence of thrombocytopenia ranges from 4.52% to 8% (11–13). The clinical implications of this complication are profound: severe thrombocytopenia not only markedly increases the risk of spontaneous bleeding but also frequently necessitates dose reductions of chemotherapeutic agents, as well as interruptions or delays in radiotherapy. Consequently, this may compromise local tumor control rates and negatively impact overall patient survival.

Thrombocytopenia, a remarkable issue resulting from radical chemoradiotherapy for NPC, adversely affects patient outcomes and quality of life. This condition primarily arises from reduced platelet production in the bone marrow and increased platelet destruction (14). In severe cases, patients may experience mucosal bleeding from the nasal passages, oral cavity, and gastrointestinal tract (15). A diminished platelet count significantly increases the risk of uncontrolled or prolonged bleeding, emphasizing the need for meticulous treatment planning and monitoring to mitigate these risks. Moreover, managing thrombocytopenia is critical, as it can lead to treatment delays and adversely impact survival outcomes. Evidence suggests that thrombocytopenia affects the timing and efficacy of subsequent therapeutic interventions, highlighting the importance of effective management strategies during and after chemoradiotherapy (16).

Therefore, precise identification of high-risk populations for developing thrombocytopenia, along with the implementation of early interventions, is essential for optimizing treatment management in patients with NPC. However, there is a notable deficiency in current research regarding large-sample risk prediction models tailored specifically to the NPC population, particularly those models that incorporate predisposing factors such as smoking history and baseline hematologic parameters. This study seeks to identify pertinent risk factors for thrombocytopenia in NPC patients undergoing chemoradiotherapy through retrospective analysis, thereby providing empirical evidence to inform clinical risk stratification and the development of individualized treatment strategies.

## 2 Materials and methods

### 2.1 Patients and data collection

This study enrolled patients initially diagnosed with NPC who underwent radical chemoradiotherapy at the Second Affiliated Hospital of Fujian Medical University from 01/01/2013 to 31/05/2024. In our study, the operational definition of radical chemoradiotherapy is based on the concurrent satisfaction of two specific criteria: (i) the delivery of a radical dose of radiotherapy, typically ranging from 66 to 70 Gy, to the primary nasopharyngeal site and cervical lymph nodes, accompanied by concurrent platinum-based chemotherapy. Specifically, all enrolled patients received a standardized chemotherapy regimen consisting of cisplatin at a dosage of 100 mg/m² every three weeks. Radiotherapy was uniformly administered using intensity-modulated radiotherapy (IMRT), with a total dose of 66 to 70 Gy delivered in 33 fractions to the primary tumor and involved lymph nodes. (ii) the execution of local interventions designed to achieve radical cure for all identified distant metastases. These interventions may include stereotactic radiotherapy or radical radiotherapy for oligometastatic lesions, such as those located in the bone, lung, and liver, or surgical resection for resectable solitary metastases. Within our cohort, these criteria are reflected in the multidisciplinary team discussions documented in the patients’ medical records, which categorize the treatment approach as ‘suitable for radical treatment’ or with the ‘treatment goal of achieving disease-free status’. The exclusion criteria included (i) presence of other malignant tumors, (ii) hematological disorders or conditions affecting coagulation, (iii) use of oral medications that impact platelet function, (iv) incomplete data, (v) pre-treatment platelet counts below 100 × 10^9^/L, and (vi) withdrawal or death during treatment. All participants received comprehensive exams to assess tumor extent, cervical lymph node involvement, and distant metastasis, with TNM staging based on the 8^th^ Edition of the American Joint Committee on Cancer criteria. Comprehensive baseline data were collected through the Second Affiliated Hospital of Fujian Medical University’s electronic medical record system, documenting variables such as sex, age, presence of hypertension and diabetes, smoking and alcohol use, chemotherapy regimen, and TNM stages.

Upon admission, fasting venous blood samples were analyzed using an XN-1000 hematology analyzer (Hessenmeikang, Japan) to assess blood cell parameters, including hemoglobin, platelets, white blood cells, neutrophils, lymphocytes, and monocytes, both before and after treatment. Platelet counts were systematically monitored, recording nadir values during treatment. Platelet counts were evaluated based on two primary criteria: (i) Prespecified time points: Peripheral blood samples were collected for complete blood count analysis prior to the initiation of treatment (baseline) and upon the completion of the entire treatment regimen. (ii) Nadir during treatment: The lowest platelet count recorded for each patient throughout the treatment period (from initiation to completion) was documented to assess the most severe hematologic toxicity. Consequently, the reported incidence of thrombocytopenia in this study comprehensively reflects platelet reductions observed either at prespecified time points or at any point during the treatment process. The normal reference range for platelet count was established as 100–300 ×10^9^/L. Thrombocytopenia was defined as a platelet count below this range, with severity graded according to the Common Terminology Criteria for Adverse Events (CTCAE) as follows: Grade I (75–100 × 10^9^/L), Grade II (50–75 × 10^9^/L), Grade III (25–50 × 10^9^/L), and Grade IV (<25 × 10^9^/L). The patients were categorized into three groups based on the reduction in platelet count: Group A (no reduction), Group B (Grade I and II thrombocytopenia), and Group C (Grade III and IV thrombocytopenia).

### 2.2 Ethics

This retrospective study was conducted in accordance with the latest version of the Declaration of Helsinki and followed established clinical practice guidelines. The study received approval from the Medical Ethics Committee of the Second Affiliated Hospital of Fujian Medical University, and the requirement for written informed consent was waived due to the retrospective nature of the study (No. 691, 2024).

### 2.3 Statistical analysis

The distribution of variables was assessed using the Kolmogorov–Smirnov test. Quantitative data conforming to a normal distribution are presented as mean ± standard deviation ( $\bar{x}$ ± s). Comparisons between groups were performed using the independent-samples t-test. Non-normally distributed measurement data are displayed as median (M) and interquartile range (Q1, Q3). Regarding non-normal data, intergroup comparisons and *post hoc* pairwise analyses were conducted using the Mann–Whitney U test. Qualitative data are summarized as frequencies and analyzed using the chi-square test or Fisher’s exact test, as appropriate. Logistic regression analysis was employed to identify risk factors for thrombocytopenia following radical chemoradiotherapy in patients with NPC. All statistical analyses were performed using SPSS version 26.0 (IBM, Armonk, NY, USA); a *p*-value of less than 0.05 was considered statistically significant.

## 3 Results

### 3.1 Comparative analysis of baseline characteristics of patients

**Figure 1:**
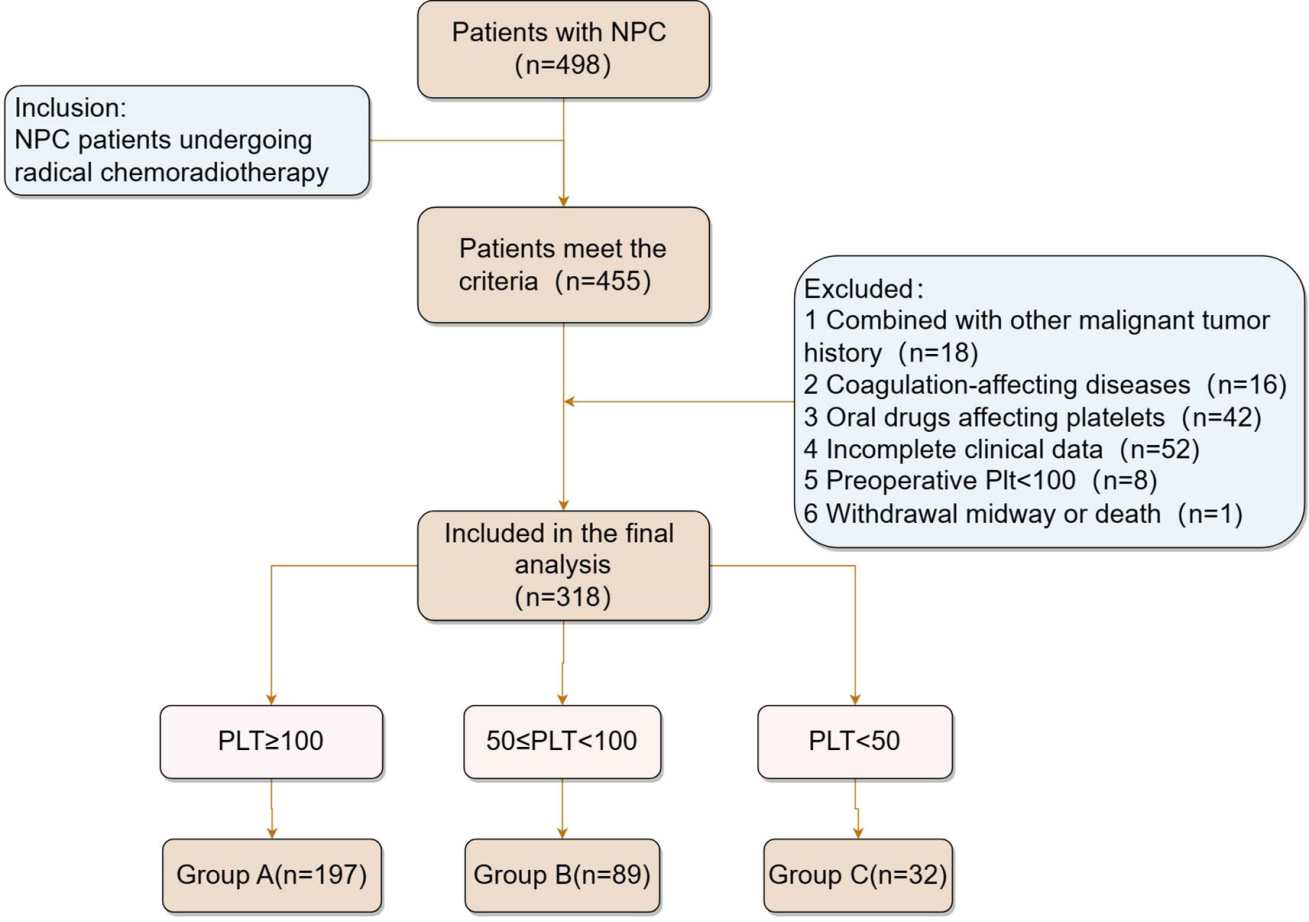
Inclusion and exclusion criteria for patients with NPC. PLT (×10^9^/L). NPC, nasopharyngeal cancer.

We retrospectively evaluated 318 patients diagnosed with NPC, comprising 220 men (69.18%) and 98 women (30.82%). Based on thrombocytopenia severity after radical chemoradiotherapy, the patients were categorized into three groups: Group A (n = 197), Group B (n = 89), and Group C (n = 32), as illustrated in **Figure 1**. A comparative analysis of platelet counts before and after treatment showed a significant difference (Z = −15.421, *p* < 0.001). Baseline characteristics, including smoking status, differed significantly across the groups (χ^2^ = 17.44, *p* < 0.001), as detailed in **Table 1**. However, no significant differences were found in age, sex, hypertension, diabetes, alcohol consumption, or TNM stages among the groups (*p*> 0.05).

**Table 1:** Baseline data of patients with NPC.

Variable	Overall N = 318^1^	Group A N = 197 (62%)^1^	Group B N = 89 (28%)^1^	Group C N = 32 (10%)^1^	χ2/F	p-value^2^
Gender					2.221	0.329
Man	220 (69.18%)	131 (66.50%)	67 (75.28%)	22 (68.75%)		
Women	98 (30.82%)	66 (33.50%)	22 (24.72%)	10 (31.25%)		
Age(years)	51.93 ± 12.43	50.98 ± 12.31	52.35 ± 12.85	56.63 ± 11.13	F=2.939	0.054
Hypertension					3.105	0.212
No	261 (82.08%)	156 (79.19%)	78 (87.64%)	27 (84.38%)		
Yes	57 (17.92%)	41 (20.81%)	11 (12.36%)	5 (15.63%)		
Diabetes					0.237	0.888
No	289 (90.88%)	178 (90.36%)	82 (92.13%)	29 (90.63%)		
Yes	29 (9.12%)	19 (9.64%)	7 (7.87%)	3 (9.38%)		
Smoking					17.44	<0.001
No	251 (78.93%)	169 (85.79%)	57 (64.04%)	25 (78.13%)		
Yes	67 (21.07%)	28 (14.21%)	32 (35.96%)	7 (21.88%)		
Alcohol					1.381	0.501
No	291 (91.51%)	183 (92.89%)	79 (88.76%)	29 (90.63%)		
Yes	27 (8.49%)	14 (7.11%)	10 (11.24%)	3 (9.38%)		
Tstage					8.531	0.202
T1	31 (9.75%)	23 (11.68%)	7 (7.87%)	1 (3.13%)		
T2	71 (22.33%)	44 (22.34%)	20 (22.47%)	7 (21.88%)		
T3	151 (47.48%)	95 (48.22%)	44 (49.44%)	12 (37.50%)		
T4	65 (20.44%)	35 (17.77%)	18 (20.22%)	12 (37.50%)		
Nstage					5.355	0.499
N0	6 (1.89%)	4 (2.03%)	2 (2.25%)	0 (0.00%)		
N1	31 (9.75%)	22 (11.17%)	8 (8.99%)	1 (3.13%)		
N2	196 (61.64%)	122 (61.93%)	56 (62.92%)	18 (56.25%)		
N3	85 (26.73%)	49 (24.87%)	23 (25.84%)	13 (40.63%)		
Mstage					0.598	0.742
M0	298 (93.71%)	185 (93.91%)	84 (94.38%)	29 (90.63%)		
M1	20 (6.29%)	12 (6.09%)	5 (5.62%)	3 (9.38%)		

^1^n (%); Mean (SD).

^2^Pearson’s Chi-squared test; One-way ANOVA; Fisher’s Exact Test for Count Data with simulated p-value.

NPC, nasopharyngeal cancer.

### 3.2 Laboratory examination of patients before chemoradiotherapy

A comparative analysis of hematological parameters among the three patient cohorts prior to radical chemoradiotherapy revealed significant differences in platelet (Z = 17.939, *p* < 0.001) and lymphocyte counts (Z = 6.729, *p* < 0.035). Additionally, correlation analysis indicated a positive relationship between platelet and lymphocyte counts (r = 0.2135, *p* < 0.001). In contrast, no significant differences were observed in hemoglobin levels or in counts of white blood cells, neutrophils, and monocytes across the groups.

### 3.3 Comparative analysis of laboratory parameters across the NPC groups

We conducted a comparative analysis of blood cell parameters across different T, N, and M stages of NPC. Significant variations were found in platelet (Z = 22.07, *p* < 0.001), white blood cell (Z = 13.732, *p* = 0.003), neutrophil (Z = 8.346, *p* = 0.039), lymphocyte (Z = 10.666, p = 0.014), and monocyte (Z = 8.386, *p* = 0.039) counts among the T stages (T1, T2, T3, and T4). Likewise, significant differences were observed in platelet (Z = 16.829, *p* < 0.001), white blood cell (Z = 8.519, p = 0.036), lymphocyte (Z = 13.054, *p* = 0.005), and monocyte (Z = 9.613, p = 0.022) counts across the N stages (N0, N1, N2, and N3). Moreover, platelet counts showed significant differences between the M stages (M0, M1; Z = −2.285, *p* = 0.022). These results highlight the substantial variations in hematological parameters across the T, N, and M stages of NPC, with detailed data presented in **Table 2**.

**Table 2:** Comparative analysis of laboratory indexes of NPC in different groups.

Variable	HGB(g/L)	PLT(×10^9^/L)	WBC(×10^9^/L)	NE(×10^9^/L)	Ly(×10^9^/L)	MO(×10^9^/L)
T						
T1	144 (135,155)	217 (183,251)	5.6(4.8,6.66)	3.5(3,4.7)	1.49(1.03,1.9)	0.4(0.3,0.5)
T2	142(128,153)	267(223,313)	6.7(5.4,7.9)	4.03(3.34,5.1)	1.65(1.24,2.04)	0.43(0.32,0.58)
T3	145(133,156)	276(233,315)	6.75(5.6,8)	4.3(3.4,5.47)	1.7(1.35,2.1)	0.46(0.38,0.6)
T4	143(132,154.5)	272(224,318)	6.9(5.89,8.65)	4.4(3.51,5.75)	1.84(1.4,2.26)	0.5(0.4,0.64)
Z	2.24	22.07	13.732	8.346	10.666	8.386
p-value	0.524	<0.001	0.003	0.039	0.014	0.039
N						
N0	139.5(115.75,148.75)	212(196.75,314)	5.505(3.99,7.578)	3.7(2.5725,6.23)	1.045(0.4175,1.375)	0.455(0.925,0.7175)
N1	144(128,151)	251(211,308)	6.66(5.31,7.64)	3.99(3.17,5.1)	1.66(1.35,2.2)	0.39(0.28,0.58)
N2	144(133,156)	256.5(221.25,302.25)	6.55(5.4,7.888)	4.165(3.3175,5.33)	1.63(1.3,2.0075)	0.455(0.36,0.5575)
N3	143(131.5,153.5)	295(254,331)	6.96(5.99,8.55)	4.46(3.675,5.48)	1.87(1.4, 2.21)	0.5(0.4,0.6)
Z	2.781	16.829	8.519	3.874	13.054	9.613
p-value	0.427	0.001	0.036	0.275	0.005	0.022
M						
M0	144(133,155)	266(223,310)	6.665(5.5,7.885)	4.195(3.3475,5.3)	1.66(1.3,2.1025)	0.47(0.36,0.6)
M1	145(128.25,153.75)	300.5(249,362.75)	6.955(6.428,9.98)	4.69(3.525,6.8575)	1.9(1.515,2.1925)	0.495(0.3975,0.6075)
Z	-0.534	-2.285	-1.652	-1.271	-1.601	-1.032
p-value	0.593	0.022	0.099	0.204	0.109	0.302

HGB, haemoglobin; PLT, platelet count; WBC, white blood cells; NE, neutrophils, Ly, lymphocytes; MO, monocytes; NPC, nasopharyngeal cancer.

The Z value is derived from the non-parametric rank sum test.

### 3.4 Risk factors for thrombocytopenia in patients undergoing radical chemoradiotherapy

We conducted a logistic regression analysis to investigate the association between thrombocytopenia (Yes vs. No) and various factors in patients with NPC following CCRT. This study identified smoking (odds ratio [OR] = 2.871, 95% confidence interval [CI]: 1.652–4.988, *p* < 0.001), preoperative platelet count (OR = 0.992, 95% CI: 0.989–0.996, *p* < 0.001), and lymphocyte count (OR = 0.651, 95% CI: 0.441–0.96, *p* = 0.03) as significant risk factors for thrombocytopenia in patients following radical chemoradiotherapy. Variables that were significant from the univariate analysis were fitted in the multivariate analysis. The multivariate analysis revealed that smoking (OR = 3.393, 95% CI: 1.88–6.123, *p* < 0.001) and preoperative platelet count (OR = 0.993, 95% CI: 0.989–0.997, *p* < 0.001) are independent risk factors for thrombocytopenia in this patient group following treatment. To further validate these findings, we categorized patients into three groups based on the severity of thrombocytopenia for an ordered logistic regression analysis: Group A (no reduction), Group B (Grade I and II thrombocytopenia), and Group C (Grade III and IV thrombocytopenia). This analysis reaffirmed smoking as an independent risk factor for thrombocytopenia (OR = 0.778, 95% CI: 0.623-0.971, *p* < 0.026) in this patient population post-treatment. Comprehensive data are provided in **Table 3** and **Supplementary Table 1** in the **Supplementary Material**.

**Table 3:** Risk factors of thrombocytopenia in patients with NPC undergoing radical chemoradiotherapy.

Variable	Univariate analysis		Multivariate analysis	
	OR(95%CI)	p-value	OR(95%CI)	p-value
Gender		0.187		
Man				
Women	0.714(0.433-1.177)			
Age(years)	1.016(0.998-1.035)	0.083		
Hypertension		0.089		
No				
Yes	0.58(0.309-1.087)			
Diabetes		0.678		
No				
Yes	0.844(0.379-1.881)			
Smoking		<0.001		<0.001
No				
Yes	2.871(1.652-4.988)		3.393(1.88-6.123)	
Alcohol		0.262		
No				
Yes	1.573(0.713-3.472)			
Metastasis		0.81		
No				
Yes	1.233(0.222-6.836)			
T stage		0.288		
T1				
T2	1.764(0.692-4.5)			
T3	1.695(0.71-4.044)			
T4	2.464(0.962-6.313)			
N stage		0.619		
N0				
N1	0.818(0.127-5.288)			
N2	1.213(0.217-6.787)			
N3	1.469(0.255-8.465)			
M stage		0.853		
M0				
M1	1.091(0.433-2.752)			
HGB(g/L)	1.008(0.994-1.023)	0.268		
PLT(×10^9^/L)	0.992(0.989-0.996)	<0.001	0.993(0.989-0.997)	<0.001
WBC(×10^9^/L)	1.021(0.983-1.061)	0.282		
NE(×10^9^/L)	0.926(0.812-1.056)	0.25		
Ly(×10^9^/L)	0.651(0.441-0.96)	0.03	0.681(0.448-1.035)	0.072
MO(×10^9^/L)	0.587(0.169-2.041)	0.403		

HGB, haemoglobin; PLT, platelet count; WBC, white blood cells; NE, neutrophils; Ly, lymphocytes; MO, monocytes.

Model Information:.

Outcome: The patients were categorized into two groups based on the reduction in platelet count (Yes vs. No) following CCRT.

## 4 Discussion

Radical chemoradiotherapy is the primary treatment modality for NPC and has demonstrated exceptional efficacy. However, both radiotherapy and chemotherapy are associated with adverse effects on bone marrow function, potentially resulting in anemia, neutropenia, and thrombocytopenia. Radiotherapy frequently leads to myelosuppression, adversely affecting platelet production. The combination of radiotherapy with chemotherapy, particularly platinum-based agents, can exacerbate thrombocytopenia in patients with NPC (17). Thrombocytopenia development during chemoradiotherapy is influenced by multiple factors, including radiation dose, type of chemotherapeutic agent, and individual patient characteristics (2, 18). If not managed promptly, a severe reduction in platelet count increases the risk of significant bleeding (19). Our study demonstrated a marked reduction in platelet count among patients with NPC following radical chemoradiotherapy (Z = −15.421, *p* < 0.001).

Chemotherapy-induced thrombocytopenia is a common hematological toxicity associated with prolonged chemotherapy. The use of induction chemotherapy is often limited by its adverse effects, including increased systemic toxicity, which can compromise subsequent concurrent chemoradiotherapy (20). Previous studies have identified various risk factors for chemotherapy-induced thrombocytopenia, including tumor type and stage (21, 22). Research as early as 1960 established a correlation between platelets and tumors. Tumor cells secrete platelet agonists that induce platelet aggregation and stimulate cytokine production, ultimately leading to increased platelet counts (23). Platelets play a crucial role in the intermediate stages of tumor progression. In this study, we analyzed blood cell parameters in patients with NPC before radical chemoradiotherapy. The results revealed significant variations in platelet counts across different T, N, and M stages of NPC, indicating an increase in platelet counts as the disease progressed.

The incidence of NPC is 2–3 times higher in men than in women, with the peak incidence occurring in individuals aged 50–59 years (24). Our study cohort comprised 220 male and 98 female patients, indicating a male-to-female ratio of approximately 2.24:1. Although the exact etiology of NPC remains unclear, it has been linked to factors such as alcohol consumption, dietary habits, and smoking (25, 26). Additionally, the overall incidence of thrombocytopenia in patients undergoing radical chemoradiotherapy for NPC may be influenced by various clinical factors, including baseline health status. Our comparative analysis indicated that smoking not only contributes to the development of NPC but also leads to a reduction in platelet count following treatment. This aligns with findings from Elkhalifa et al. (27), who demonstrated that tobacco snuff use decreases platelet counts in adult Wistar rats. Further research has identified a negative correlation between the number of pack-years smoked and platelet count in smokers, suggesting that prolonged smoking exacerbates this effect, typically resulting in lower platelet counts (28).

The reduction in platelet count may result from the direct impact of nicotine on oxidative stress, which contributes to thrombocytopenia (28). Smoking has been associated with increased platelet aggregation and enhanced blood coagulation, promoting platelet activity and further exacerbating the condition. Our univariate analysis revealed that patients with NPC who smoked experienced a 2.871-fold greater reduction in platelet count following radical chemoradiotherapy than that experienced by non-smoking patients (OR = 2.871, 95% CI: 1.652–4.988, *p* < 0.001). Subsequent multivariate analysis confirmed that the platelet count in smoking patients was 3.393 times lower than that in non-smoking patients following treatment (OR = 3.393, 95% CI: 1.88–6.123, *p* < 0.001). Smoking was identified as an independent risk factor for the reduction in platelet count after radical chemoradiotherapy in patients with NPC.

**Figure 2:**
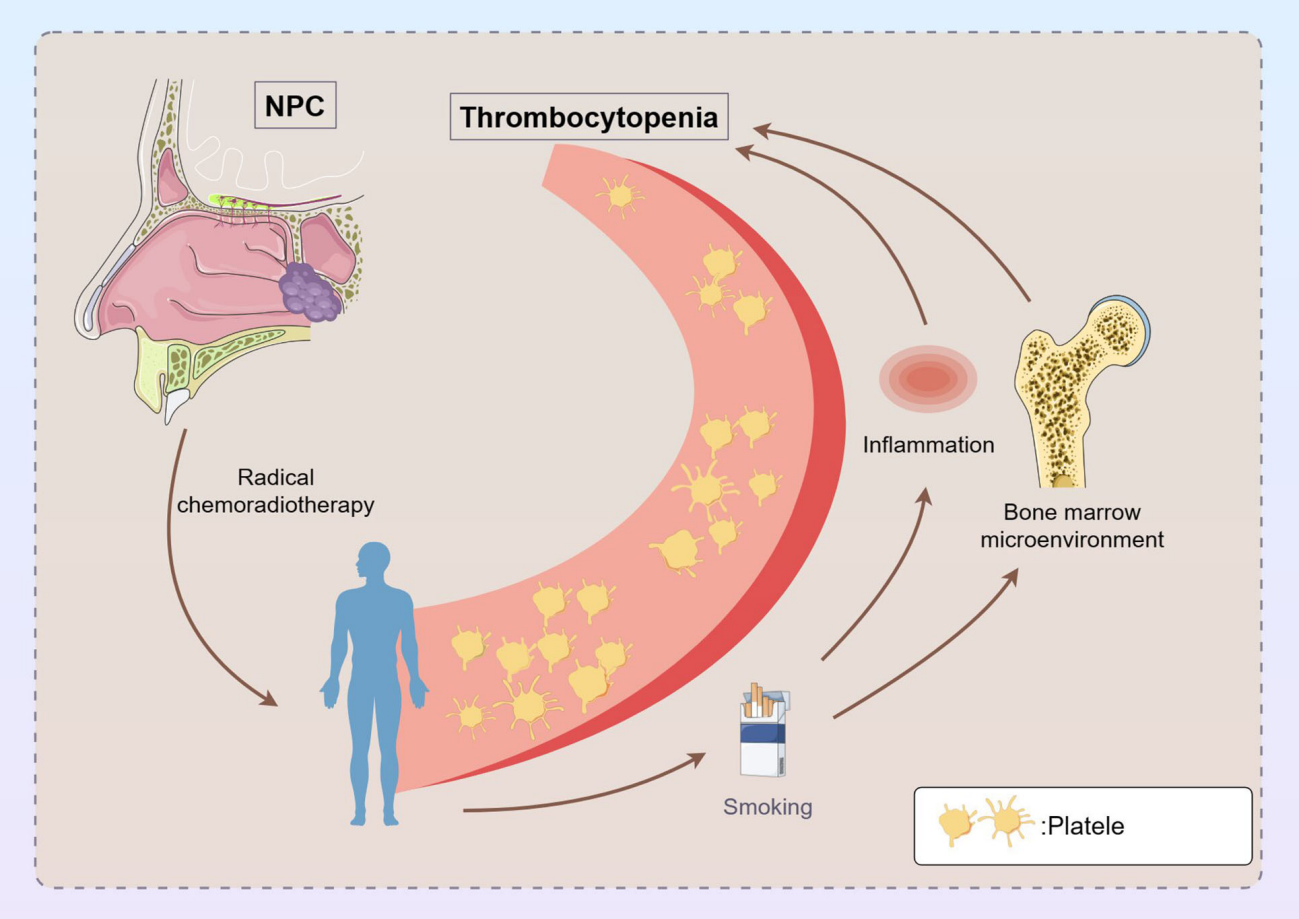
Mechanism underlying the effect of smoking on thrombocytopenia in patients with NPC undergoing radical chemoradiotherapy. In patients with NPC, smoking may affect the treatment outcome by initiating inflammation, damaging the surrounding tissues, and interfering with tumor irradiation during radiation therapy. Smoking may lead to changes in the bone marrow microenvironment, which affects megakaryocyte differentiation and platelet generation. NPC, nasopharyngeal cancer.

Patients who smoke experience more severe thrombocytopenia after radical radiotherapy and chemotherapy compared with non-smokers. This phenomenon may be attributed to the various effects of smoking on the body. Research indicates that smoking causes hematological changes and adversely affects treatment response and recovery in cancer patients (29). Smoking impacts treatment outcomes by inducing inflammation, damaging surrounding tissues, and interfering with tumor irradiation during radiation therapy (30). Specifically, it may alter the bone marrow microenvironment, affecting megakaryocyte differentiation and platelet production (31). Additionally, smokers face a high risk of complications, such as infections and other blood-related disorders. Studies show that smokers undergoing chemotherapy report a high symptom burden, including fatigue and pain, which may exacerbate thrombocytopenia (32). Smoking may also alter drug metabolism, influencing the metabolism of chemotherapeutic drugs and affecting their efficacy and side effects (33). Furthermore, smoking can weaken the immune system, increasing susceptibility to infections during treatment, which can further reduce platelet levels. **Figure 2** illustrates the mechanism by which smoking exacerbates thrombocytopenia in patients with NPC undergoing radical chemoradiotherapy. Interventions targeting smoking cessation are crucial for improving prognosis and quality of life during treatment (34, 35). In conclusion, the effects of smoking on cancer patients are multifaceted, aggravating thrombocytopenia during radiotherapy and chemotherapy, thus highlighting the need for clinicians to consider smoking history in treatment planning and to provide effective smoking cessation support.

In clinical practice, delayed intervention in cases of hemorrhage caused by NPC can be life-threatening. The causes of significant hemorrhage in patients with NPC include not only tumor invasion of blood vessels but also bone marrow suppression following radiotherapy and chemotherapy. This suppression exacerbates thrombocytopenia and increases the risk of bleeding. Study shows radiation therapy, with or without chemotherapy, leads to a marked reduction in lymphocyte counts due to their heightened sensitivity to chemoradiotherapy (36). The initial analysis in this study indicated a potential association between lymphocyte count and platelet count. However, subsequent multivariate analysis revealed that this association was not independent. Clinically, this implies that variations in platelet counts cannot be solely inferred or predicted based on lymphocyte counts. The alteration in lymphocyte count may merely represent a concomitant phenomenon within a broader systemic pathological process, rather than serving as a direct causative factor influencing changes in platelet count. Consequently, in this study cohort, lymphocyte count does not serve as an independent predictor of platelet count.

In the context of NPC, a reduced platelet count complicates treatment planning, as chemotherapy is expected to further decrease platelet levels. Multivariate analysis identified a low initial platelet count as an independent risk factor for thrombocytopenia following radical chemoradiotherapy in patients with NPC. Significantly reduced platelet levels increase the risk of tissue hemorrhage and often necessitate chemotherapy dosage reductions, potentially leading to treatment failure (37). Maintaining a platelet count above 80 × 10^9^/L is generally considered a prerequisite in patients with NPC to undergo radiotherapy and chemotherapy. During chemotherapy, managing severe thrombocytopenia is essential and involves interventions such as dose reductions for radiotherapy and chemotherapy, treatment postponement, platelet transfusions, and the administration of recombinant human thrombopoietin and recombinant human interleukin-11 (28). Both of these agents have been approved by the National Medical Products Administration for the treatment of thrombocytopenia induced by chemotherapy or chemoradiotherapy.

This study has some limitations. First, this study is that treatment intent was determined based on retrospective medical record review. Although we applied the above strict criteria, some degree of classification bias may still exist compared to prospectively defined standardized assessments in prospective studies. Second, the single-center design limits its generalizability, as all samples were obtained exclusively from the Second Affiliated Hospital of Fujian Medical University. The influence of regional variations and potential confounding factors cannot be overlooked. Further research is needed to validate the applicability of these findings to other regional populations. As a result, we were unable to assess the differential effects of specific chemotherapeutic agents on platelet count. Smoking and low platelet counts were independent risk factors for thrombocytopenia following radical chemoradiotherapy in patients with NPC. Dynamic monitoring for platelet levels in patients who smoke or have low initial counts is crucial during the course of treatment. Timely adjustments to treatment regimens and the provision of supportive care are essential. Comprehensive management and preventive strategies can enhance therapeutic outcomes, mitigate the adverse effects of treatment, and improve the quality of life for patients with NPC.

## Data Availability

The original contributions presented in the study are included in the article/**Supplementary Material**. Further inquiries can be directed to the corresponding authors.

## Supplementary material

Supplementary Table 1 Ordinal logistic regression analysis of factors associated with thrombocytopenia (N = 318).
