# Supplementary material for: Identifying key risk factors for thrombocytopenia in patients undergoing radical chemoradiotherapy for nasopharyngeal cancer: a retrospective study
Source: Front Oncol. 2026 Mar 2;16:1752387. doi: 10.3389/fonc.2026.1752387 (PMC12989368; doi:10.3389/fonc.2026.1752387)
Supplement: Supplementary Table 1 — Ordinal logistic regression analysis of factors associated with thrombocytopenia (N = 318). [file Table1.docx]

Identifying key risk factors for thrombocytopenia in patients undergoing radical chemoradiotherapy for nasopharyngeal cancer: A retrospective study

Qiongling Huang^1^, Xinyuan Luo^1^, Baoling Li^1^, Ming Lu^1^, Xiaofang Chen^1*^, Enhui Qiu^1*^

Table S1. Ordinal logistic regression analysis of factors associated with thrombocytopenia (N=318)

| **Variable** | **β (SE)** | **p-value** | **OR (95% CI)** |
| --- | --- | --- | --- |
| **Demographics** |  |  |  |
| Gender(Male vs. Female) | -0.036(0.099) | 0.719 | 0.965(0.793,1.173) |
| Age (per 1-year increase) | 0.005(0.003) | 0.092 | 1.005(0.999,1.012) |
| **Clinical Factors** |  |  |  |
| Hypertension(Yes vs. No) | 0.107(0.104) | 0.305 | 1.113(0.907,1.365) |
| Diabetes(Yes vs. No) | -0.025(0.134) | 0.85 | 0.975(0.75,1.267) |
| Smoking(Yes vs. No) | -0.251(0.113) | **0.026** | 0.778(0.623,0.971) |
| Alcohol(Yes vs. No) | 0.134(0.159) | 0.4 | 1.143(0.837,1.562) |
| T stage (Ref: T1) |  |  |  |
| T2 vs. T1 | -0.379(0.148) | **0.01** | 0.685(0.513,0.914) |
| T3 vs. T1 | -0.182(0.113) | 0.106 | 0.833(0.688,1.039) |
| T4 vs. T1 | -0.145(0.098) | 0.141 | 0.865(0.713,1.049) |
| N stage (Ref: N0) |  |  |  |
| N1 vs. N0 | -0.329(0.271) | 0.225 | 0.72(0.423,1.224) |
| N2 vs. N0 | -0.307(0.137) | **0.025** | 0.735(0.562,0.962) |
| N3 vs. N0 | -0.133(0.88) | 0.128 | 0.875(0.737,1.039) |
| M stage(M0 vs. M1) | -0.05(0.154) | 0.975 | 0.995(0.736,1.346) |
| **Laboratory** |  |  |  |
| HGB(g/L) | 0.001(0.003) | 0.74 | 1.001(0.996,1.006) |
| PLT(×10^9^/L) | -0.002(0.001) | **0.001** | 0.998(0.997,0.999) |
| WBC(×10^9^/L) | 0.003(0.005) | 0.517 | 1.003(0.993,1.014) |
| NE(×10^9^/L) | 0.023(0.024) | 0.347 | 1.023(0.975,1.073) |
| Ly(×10^9^/L) | -0.01(0.006) | 0.07 | 0.99(0.979,1.001) |
| MO(×10^9^/L) | -0.273(0.229) | 0.233 | 0.761(0.486,1.192) |

Model Information:

Outcome: The patients were categorized into three groups based on the reduction in platelet count: Group A (no reduction), Group B (Grade I and II thrombocytopenia), and Group C (Grade III and IV thrombocytopenia).

Test of Parallel Lines (Proportional Odds):χ² = 17.964, df=19, *p*=0.525. The assumption was met.
